# Knowledge and Attitudes on the Use of Assisted Reproductive Technologies (ART) Among Women of Reproductive Age in Southeast Alabama

**DOI:** 10.7759/cureus.95243

**Published:** 2025-10-23

**Authors:** Melissa M Garcia Gonzalez, Praful G Patel, Angellar Manguvo, Benford Mafuvadze

**Affiliations:** 1 Anatomy and Molecular Medicine, Alabama College of Osteopathic Medicine, Dothan, USA; 2 Obstetrics and Gynecology, Southeast Health Medical Center, Dothan, USA; 3 Obstetrics and Gynecology, Alabama College of Osteopathic Medicine, Dothan, USA; 4 Graduate Health Professions in Medicine, University of Missouri-Kansas City School of Medicine, Kansas City, USA

**Keywords:** assisted reproductive technology (art), fertility, in vitro fertilization (ivf), reproduction, women’s health

## Abstract

Background: Assisted reproductive technologies (ARTs), including in vitro fertilization (IVF), offer solutions to infertility, yet public understanding and acceptance vary widely. In Southeast Alabama, recent legal developments following the Alabama Supreme Court ruling giving frozen embryos similar rights to children have intensified debates around ART. This study aimed to assess the knowledge, attitudes, and perceptions of women of reproductive age regarding ART and explore how demographic and experiential factors influence these views.

Methodology: A cross-sectional survey was conducted among 144 women aged 16-45 residing in Southeast Alabama. The survey instrument, developed through literature review and expert consultation, included sections on demographics, knowledge, and attitudes toward ART. Data were analyzed using SPSS version 30 (IBM Corp., Armonk, NY), employing descriptive statistics, t-tests, analysis of variance (ANOVA), and chi-square tests to identify significant associations.

Results: Most participants (*n* = 128, 89%) reported moderate familiarity with ART, though specific knowledge gaps were evident. For example, only (*n *= 37, 26%) of participants correctly identified IVF success rates for women over 40. While most of the women supported egg (*n *= 120, 83%) and sperm (*n* = 122, 85%) donation, fewer were comfortable with personal involvement in donation or surrogacy. Fertility issues and cost were the most cited factors influencing ART decisions. Support for federal funding was highest for individuals with medical infertility, with limited support for lifestyle-related delays. Christian participants and rural residents showed lower support for ART, while non-White and more educated women expressed greater acceptance. Most participants favored scientific consensus over religious or political bodies in determining the legal status of frozen embryos.

Conclusions: Women in Southeast Alabama generally support ART but hold nuanced views shaped by personal, cultural, and socioeconomic factors. Knowledge gaps and ethical concerns on ART persist among women, underscoring the need for targeted education and policy efforts to improve informed decision-making and equitable access to fertility care.

## Introduction

It is estimated that around 9% of men and about 11% of women of reproductive age in the United States experience fertility problems at some point [1]. Assisted reproductive technologies (ART), such as in vitro fertilization (IVF), provide an option that enables many people facing fertility challenges to have their own biological children. According to statistical data released by the Centers for Disease Control and Prevention (CDC), in 2022 alone, 98,289 babies were born using ART in the United States [2]. Advancements in ART have led to its rise in popularity, wider acceptance, and increased utilization. This use of advanced technology has enabled physicians to perform complex manipulations such as combining gamete parts of three individuals in mitochondrial replacement therapy [3] and even have the capacity to make transferable genomic alterations of embryos [4]. While some of these ART procedures are now permitted in some parts of the world, they remain banned in the United States and, as such, are not part of routine medical practice [5,6].

It is clear that while the general use of ART is widely accepted, there continue to be divided opinions on the acceptability of ART among people in different societies. For example, while there currently appears to be a worldwide consensus that some forms of ART, such as the use of reproductive cloning in humans, are not ethically acceptable [6], there is an indication that most people could accept genetic alterations of human embryos to correct common monogenic mutation disorders such as sickle cell anemia. There is a worldwide objection to the use of ART for the enhancement or selection of perceived desirable human qualities such as intelligence or beauty [6]. The recent judgment by the Alabama Supreme Court declaring that frozen embryos have similar rights as children brought another dimension concerning legalities in the use of ART in humans [7]. In light of this recent judgment, the American College of Obstetrics and Gynecologists and several other groups issued statements condemning this ruling, highlighting the legal burden that the ruling would bring to physicians assisting families with ART. Conversely, this judgment was well received in some communities as a true demonstration of respecting the fact that life begins at conception. These differences in opinion and views across different groups of society prompted us to investigate how knowledgeable the general women population in Alabama was on the use of ART. Previous studies have shown that despite the availability of reliable sources of information on ART in humans, there is a general lack of knowledge and lots of misperceptions on ART among the general population [8,9].

We believe that societal perspectives on controversial issues, such as the use of some forms of ART, are subject to continual evolution and may change across generations. Consequently, it is essential that we periodically survey diverse populations to evaluate both their knowledge and opinions. For example, as previously stated, while certain aspects of ART, such as genetically engineering embryos, are presently deemed ethically unacceptable [6], there is a potential for societal shift over time, especially if used to address monogenic disorders, possibly resulting in broader acceptance of these practices. Given that women alone undergo the experience of carrying a fetus, it is essential to capture their perspectives on matters that directly shape reproductive health, including the use of ART. This study assessed the knowledge and opinions of women of reproductive age regarding ART. In addition, the study examined how variables such as education level, socioeconomic status, and religious beliefs influenced their perceptions and attitudes toward the use of ART for conception.

We reckon that assessing women’s understanding of fertility-related topics, including ART, allows for the identification of opportunities to improve the dissemination of accurate information. The study provided insight into the complexities surrounding ART and highlighted some of the factors that influence its acceptance and utilization among women. Overall, the study provides information that healthcare providers can use to formulate effective strategies for communicating ART-related information to women dealing with fertility challenges.

## Materials and methods

Research questions

This research employed a survey design to evaluate women’s knowledge, attitudes, and opinions on ART. The study assessed the level of knowledge among women in Southeast Alabama regarding ART, explored their prevailing attitudes and opinions concerning the ethical, social, and financial implications of ART, and examined how demographic and experiential factors influence their understanding and perspectives on these technologies.

Instrumentation

We developed the survey instrument through a review of existing literature, informal interviews with women, and consultations with subject-matter experts. A significant number of questions were adapted from a survey previously utilized by Szalma and Bitó [8], with modifications made to align with the context of this study. The instrument underwent pilot testing, which informed revisions to enhance its validity. The final version of the questionnaire comprised three sections, described in detail below (Appendix).

The Demographics section consisted of nine questions aimed at collecting data on participants’ ethnicity, age, education level, religion, income level, and residential area (urban, suburban, or rural). Comprising nine core questions, the Attitudes and Perspectives section explored opinions on ART-related topics, including sperm and egg donation, IVF accessibility, federal funding for ART, and factors influencing the use of ART. Additionally, six questions examined attitudes toward the legal status of frozen embryos. Lastly, the Knowledge section included eight questions designed to assess participants’ understanding of ART. Topics covered included IVF success rates, potential risks and complications for mothers and babies, and the number of cycles typically required for successful conception.

Study population and eligibility

The study population consisted of women of reproductive age residing in Dothan, Southeast Alabama. Eligibility criteria included women aged 16-45 years, currently living in the designated region, and able to provide informed consent. Women outside this age range, those residing elsewhere, or those unable to consent were excluded. Dothan is located in a predominantly rural area surrounded by farming communities, with 19.2% of residents living below the poverty line [10]. This geographic and demographic diversity enabled the inclusion of perspectives from women across urban, suburban, and rural settings.

Sampling and data collection procedures

The target sample size for this study was calculated to ensure adequate statistical power. A priori power analysis was conducted using G*Power (version 3.1.9.7) for the primary analyses [11]. Assuming moderate effect sizes, an alpha of 0.05, and a desired power of 0.80, the minimum required sample size was calculated to be 128 participants. To account for potential incomplete surveys, we increased our sample size to 144 participants [11]. This sample size supports reliable subgroup comparisons and assessment of associations between participant characteristics and ART knowledge and attitudes.

Recruitment was conducted primarily at two OBGYN clinics in Dothan, where all eligible women seeking care were invited to participate. To broaden representation, additional recruitment was carried out at daycare centers. At both sites, a member of the research team explained the study’s purpose, procedures, and confidentiality measures before enrollment. In clinical settings, this explanation was provided by a medical student researcher or, when unavailable, by a faculty physician. At daycare centers, the medical student researcher provided the same explanation before distributing flyers with a QR code linking to the survey. Following approval by the Institutional Review Board (IRB# 24-05-06-001), data were collected through an anonymous online survey, which remained accessible for eight weeks to maximize participation.

Data analysis

Data were exported from Qualtrics and analyzed using SPSS version 30 (IBM Corp., Armonk, NY). Some groups were combined into broader categories to address limited representation within certain demographic categories. This approach facilitated meaningful statistical comparisons and minimized challenges associated with small subgroup sizes. For example, ethnicity was recoded into two binary categories: Caucasian/White and non-White. Most items on both the knowledge and attitude scales were categorical, and responses were analyzed in their original format to determine frequencies. Five items on the attitude subscale were re-coded to a 1-5 Likert scale, where higher scores indicated more positive attitudes toward ART and lower scores reflected less favorable perspectives. After data cleaning and recoding, we generated descriptive statistics to summarize participants’ demographic profiles, as well as their knowledge and attitudes toward ART. Independent t-tests and analysis of variance (ANOVA) were conducted to evaluate differences in mean scores across demographic groups for the Likert-scale attitude items. For the categorical items on both the knowledge and attitude subscales, chi-square tests were employed to assess relationships among variables, evaluating whether response patterns varied significantly across demographic groups.

## Results

The results are presented in four sections. The first section details the demographic distribution, while the second and third sections focus on attitudes and knowledge, respectively. The final section explores how perspectives and support for ART differ across demographic groups. 

Demographic characteristics of the participants 

A total of 144 women participated in the study, with the majority identifying as White. Christianity was the predominant religion, followed by those with no religious affiliation, while other religions were minimally represented. Most participants lived in rural areas, with fewer residing in suburban or urban locations. Most participants were primarily in their mid-20s to mid-30s, with smaller numbers in older age groups. In terms of childbearing, the largest group consisted of first-time pregnant women, followed by those who had never been pregnant. Educational attainment varied, with most participants having completed high school or a bachelor’s degree. Household income showed a broad distribution, with a significant proportion in lower and middle-income brackets. Table 1 provides a detailed breakdown of participants’ demographic characteristics. 

**Table 1:** Demographic and socioeconomic profiles of the study participants. GED, General Educational Development

Ethnicity	*n* (%)
Caucasian/White	105 (73%)
Non-White	39 (27%)
Religion	
Christianity	103 (72%)
Other religions	10 (7%)
No religion/atheism	31 (22%)
Age (years)	
16-24	38 (26%)
25-34	54 (38%)
35-44	24 (17%)
45	28 (19%)
Education	
No high school	4 (3%)
High school/GED	48 (33%)
Associate degree/Trade school	25 (17%)
Bachelor’s degree	49 (34%)
Graduate degree	18 (13%)
Total	144 (100%)

**Table 2:** Additional demographics and socieoeconomic profiles of study participants.

Know someone who used ART	n (%)
Yes	57 (40%)
No	87 (60%)
Rural/Urban	
Urban	19 (13%)
Suburban	43 (30%)
Rural	82 (57%)
Given birth	
Never been pregnant	38 (26%)
First pregnancy	54 (38%)
Pregnant before, but no birth	24 (17%)
Given birth before	28 (19%)
Income	
Less than $25,000	25 (17%)
$25,001-$50,000	32 (22%)
$50,001-$75,000	37 (26%)
$75,001-$100,000	18 (13%)
$100,000-$150,000	17 (12%)
>$150,000	15 (10%)
Total	144 (100%)

Attitudes toward ART 

For the categorical items on the attitude subscale, the results indicated varying levels of support and comfort regarding different aspects of ART. A significant majority expressed support for egg and sperm donation, while fewer participants felt comfortable with their partner serving as a sperm or egg donor. Similarly, fewer respondents were willing to consider having a biological child via a surrogate mother. Notably, most participants believed that the availability of IVF and other ART does not encourage delayed childbearing. Table 3 presents the mean scores for the Likert-scale attitude items.

**Table 3:** Frequency distributions of binary categorical attitudes toward assisted reproductive technologies (ART) items. IVF, in vitro fertilization

Survey item	Yes, *n* (%)	No, *n* (%)
Do you think that the availability of IVF and other ART encourages people to delay childbearing?	50 (35%)	94 (65%)
Do you support the practice of egg donation?	120 (83%)	23 (16%)
Do you support the practice of sperm donation?	122 (85%)	21 (15%)
Would you be comfortable with your partner being a sperm or egg donor?	53 (37%)	90 (63%)
Would you ever consider having your biological baby using a surrogate mother?	58 (40%)	85 (59%)

Five Likert-scale items measuring attitudes toward ART were coded and averaged to calculate each participant’s overall attitude score. The results showed strong support for the use of ART in humans. However, participants displayed moderate willingness to consider using IVF treatment and to pay for IVF services. Agreement with the legal classification of frozen embryos as “children” and support for laws granting “legal protection to embryos at all stages of development” had low levels of acceptance. Table 4 provides the mean scores for the Likert-scale attitude items.

**Table 4:** Mean scores for Likert-scale attitudes toward assisted reproductive technology (ART) items.

Attitude item	Mean score
What is your opinion on the use of ART in humans?	4.24
Would you/ Have you ever considered using IVF treatment?	2.63
Willingness to pay to have a child through in vitro fertilization	3.24
Agreement with this legal ruling that classifies frozen embryos as *children*	3.28
Support of a law that provides legal protection for embryos, whether frozen or in the uterus, at all stages of development	2.95

We asked participants to identify the factors likely to influence their decision to use or not use IVF by selecting all applicable options from the provided list. The results revealed that fertility issues and cost were the most frequently cited factors influencing decisions. Health status also perceivably played a significant role, though to a slightly lesser extent. Fewer than a third of participants identified the number of desired children and religion as key factors in their decision-making process. Table 5 presents the frequency distributions of factors influencing participants’ decisions regarding the use of IVF.

**Table 5:** Factors influencing the decision to use or not use in vitro fertilization (IVF).

Factor	*n* (%)
Fertility issues	122 (91%)
Cost	111 (83%)
Health status	84 (63%)
Number of desired children	38 (28%)
Religion	35 (26%)

We also asked participants to share their opinions regarding federal funding for IVF. Results revealed that participants most strongly supported federal funding for individuals whose fertility was compromised due to medical conditions, such as cancer treatment, or for those unable to conceive their first child naturally. There was moderate support for funding IVF for individuals pursuing delayed parenthood due to professional or national commitments, as well as for those seeking additional children. A smaller proportion of participants believed that the cost of IVF should be entirely borne by individuals or that federal funding should be prioritized for other medical issues. Table 6 presents the detailed frequency distributions of these opinions. 

**Table 6:** Circumstances when federal government funding of in vitro fertilization (IVF) is deemed appropriate.

Statement	*n* (%)
When fertility has been risked/sacrificed (e.g., due to cancer treatment)	98 (69%)
When having a first child and being unable to conceive naturally	94 (66%)
When a decision has been made to have a child later in life due to medical studies and other commitments	62 (44%)
When having another child (in addition to any they currently have) and are unable to conceive naturally	59 (42%)
When a decision has been made to have a child later in life due to medical studies and other national commitments, such as military deployments	59 (42%)
Funding for IVF should always be done by Individuals	31 (22%)
The federal government should never fund IVF because there are better medical issues to fund with taxpayers’ money	13 (9%)

Participants were asked to share their reasoning regarding court rulings and legal interventions related to the status of embryos. The results showed a variety of perspectives among participants. A sizable portion based their views on personal values and principles, while about a third expressed concerns about infringements on reproductive rights and restricted access to ART. A smaller group recognized the potential of frozen embryos for human life or supported the ruling as a means of providing legal protection for embryos. Professional expertise and religious conflicts were the least frequently cited factors. Further details on these responses are presented in Table 7. 

**Table 7:** Reasoning behind responses to the court ruling and legal interventions on embryos. ART, assisted reproductive technologies

Reason	*n* (%)
It aligns with my personal values and principles.	62 (45%)
I oppose the ruling as it infringes on reproductive rights and could restrict access to ART.	52 (37%)
I oppose the ruling as it infringes on reproductive rights and could restrict access to ART.	52 (37%)
I recognize frozen embryos have the potential for human life, influencing my perspective.	36 (26%)
I believe life begins at conception, so embryos should be recognized as children once they are fertilized.	29 (21%)
I support the ruling as it provides legal protection for embryos and potential life.	26 (19%)
I believe embryos should be recognized as children only when they have a recognizable heartbeat.	21 (15%)
I am unsure about the ruling and would like to learn more before forming an opinion.	20 (14%)
I have professional expertise in this area.	11 (8%)
It conflicts with my religious beliefs	6 (5%)

**Figure 1:**
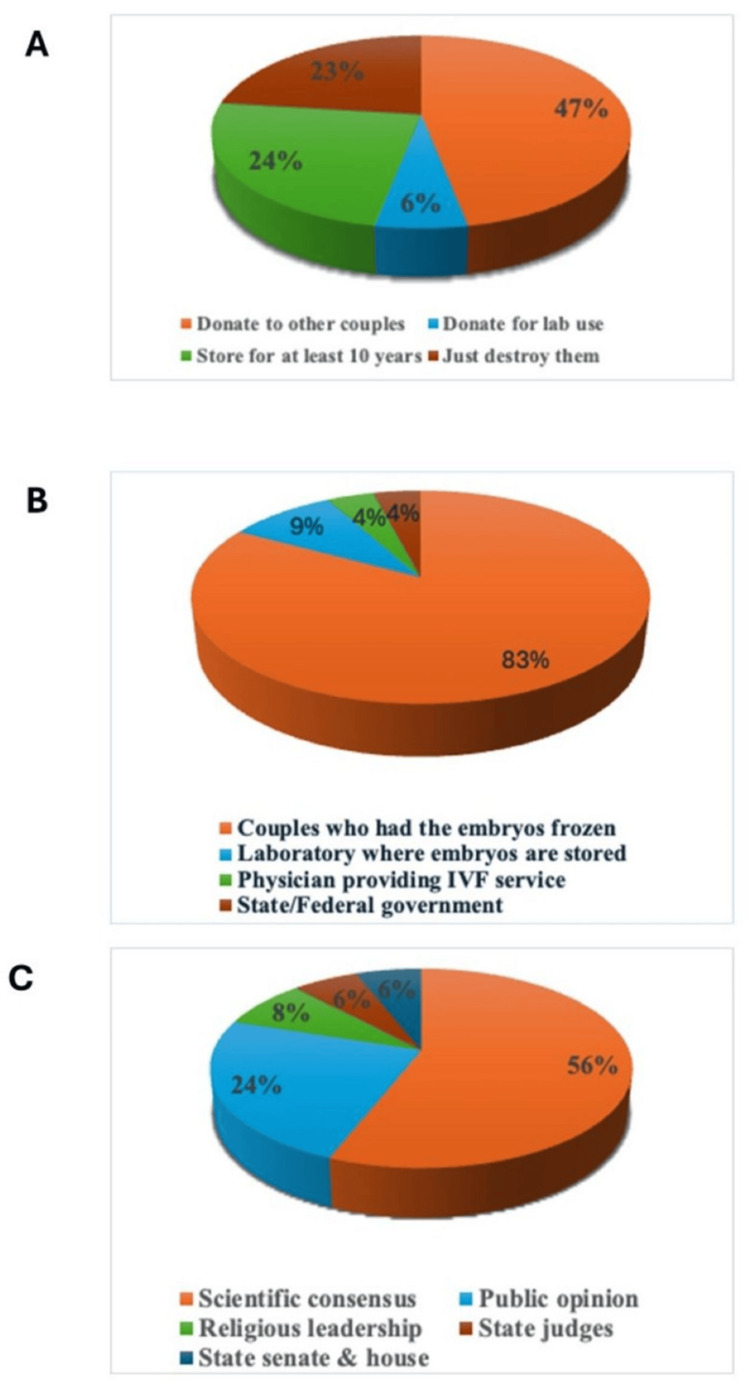
Perspectives on the disposition and governance of frozen embryos. Participants (n = 144) responded to an online survey and answered the following questions: (A) What should be done with the remaining frozen eggs? (B) Who should have legal authority over the remaining frozen embryos? and (C) Who should determine the legal status of frozen embryos?

We also asked the participants about the handling and legal considerations for frozen embryos, and to results revealed diverse opinions. Nearly half of the participants suggested donating unused embryos to other couples, while smaller proportions supported options such as storing them for a specific period, destroying them, or using them for laboratory experiments. The majority believed that couples who had the embryos frozen should hold the legal authority to decide their fate. Support for decisions being made by laboratories, physicians, or state and federal governments was minimal. Regarding the determination of the legal status of frozen embryos, scientific consensus was the most favored approach, followed by public opinion. Support for decisions led by religious leadership, state judges, or legislative bodies was notably lower. Further details of these responses are presented in Figure 1.

Knowledge of ART 

The second section of the survey assessed participants’ knowledge of issues related to ART. As an initial step, respondents were asked to self-evaluate their level of understanding of ART. A majority, 85 (59%), reported having *some knowledge*, followed by 43 (30%) who identified as *fairly knowledgeable*. Smaller proportions rated themselves as *very knowledgeable* (9, 6%) or as having *no knowledge* (7, 5%) of ART.

Building on previous research that identified an overestimation of ART efficacy, we assessed participants’ knowledge of specific ART outcomes. One question asked participants about the typical number of IVF cycles required for successful conception, with the correct answer being two to three cycles. Notably, 69 (48%) participants correctly identified this range. Another question assessed participants’ understanding of the IVF success rate for women over 40 years of age, with the correct answer being 20% or less. Only 37 (26%) participants accurately identified this figure. Nearly three-quarters overestimated the success rate, with about half of them rating it between 21% and 40%.

In further assessing participants’ knowledge of the potential risks and complications associated with ART, we explored perceptions regarding risks for both mothers and babies. Participants identified a greater number of risks and complications for mothers compared to babies. Miscarriage and ectopic pregnancy emerged as the most accurately identified risks for mothers undergoing fertility treatments. Approximately half of the participants correctly recognized physical and emotional stress, as well as multiple pregnancies, as significant risks. Premature delivery, ovarian hyperstimulation syndrome, and post-partum depression were correctly acknowledged by about one-third of respondents, while obesity was the least commonly perceived risk. All responses regarding maternal risks are accurate, except for obesity.

Approximately two-thirds of participants correctly identified birth defects and low birth weight as risks and complications to babies associated with ART. About one-third inaccurately recognized Down syndrome as a potential risk, while a smaller proportion even considered autism and allergies as concerns. Childhood obesity and attention-deficit/hyperactivity disorder (ADHD) were identified as risks by just over 14 (10%) participants. Among the perceived risks to babies, only birth defects and low birth weight are accurate; the remaining responses are incorrect. Detailed frequency distributions of these perceptions are provided in Table 8. 

**Table 8:** Perceptions of potential risks or complications for women and children conceived using assisted reproductive technologies (ART).

Potential risk or complication for women using ART	n (%)
Miscarriage	83 (87%)
Ectopic pregnancy	67 (71%)
Stress. IVF can be draining for the body, mind, and finances	70 (52%)
Multiple pregnancy	66 (49%)
Premature delivery	51 (38%)
Ovarian hyperstimulation syndrome	41 (31%)
Increased likelihood of post-partum depression	37 (28%)
Increased likelihood of obesity	20 (15%)
Perceptions of potential risks or complications for the baby	
Birth defects	77 (65%)
Low birth weight	75 (64%)
Increased likelihood of Down syndrome	34 (29%)
Increased likelihood of being born autistic	27 (23%)
Increased likelihood of developing allergies	24 (20%)
Childhood obesity	15 (13%)
Increased likelihood of being born with ADHD	13 (11%)

Demographic differences in perceptions and knowledge of ART 

ANOVA and t-test analyses of the continuous attitude items revealed no significant differences across most demographic variables, such as age, ethnicity, and education. However, a significant difference was observed based on religion. Christian participants exhibited a significantly lower cumulative attitude score (*M* = 3.11) compared to participants with no religion (*M* = 3.76) and those belonging to other religions (*M* = 3.60) (*F *= 7.226, degrees of freedom (DF) = 2, *P* < 0.001). Another significant difference was in relation to familiarity with ART. Participants who personally knew someone who had undergone ART demonstrated a higher cumulative attitude score (*M* = 3.51) compared to those who did not (*M* = 3.12) (*t* = 2.73, DF = 143, *P* < 0.05). 

We performed chi-square tests on categorical items to evaluate whether response patterns varied significantly across demographic groups. The analyses also explored variations in responses based on participants’ childbirth history and familiarity with someone who had undergone ART.

The findings revealed significant trends. Notably, we observed most differences in perceptions related to the management of remaining frozen embryos, while attitudes toward sperm and egg donation and surrogacy showed minimal variation across demographic groups.

Chi-square tests also revealed that participants coded as non-White showed more favorable attitudes toward ART compared with their White counterparts. Similarly, participants practicing non-Christian religions demonstrated greater support for ART than those identifying as Christian. Favorability toward ART was positively associated with education level, with more educated participants exhibiting greater acceptance. Geographic location also influenced attitudes, with rural residents being less favorable toward ART than those living in suburban and urban areas. No significant differences were observed in attitudes based on age. Experiential factors further shaped perceptions, as participants who personally knew someone who had undergone ART expressed more favorable attitudes than those without such connections. Additionally, individuals with a history of unsuccessful pregnancies viewed ART positively. Table 9 presents the corresponding chi-square test results for the demographic and experiential factors, along with the related categorical questions.

**Table 9:** Chi-square results for demographic and experiential factors. Educ, education level; SES, household income; K/S, knowledge of someone who has undergone ART; Birth, birth status; NS, statistically not significant *Statistically significant

	Ethnic	Religion	Age	Educ	SES	Location	K/S	Birth
Would you/Have you ever considered using IVF?	NS	NS	NS	*P* < 0.05*	NS	NS	*P* < 0.05*	NS
Do you think that the availability of IVF and other ART encourages people to delay childbearing?	*P* < 0.05*	*P* < 0.05*	NS	NS	NS	NS	NS	NS
Do you support the practice of egg donation?	NS	*P* < 0.05*	NS	NS	NS	NS	NS	NS
Do you support the practice of sperm donation?	NS	*P* < 0.05*	NS	NS	NS	NS	NS	NS
Would you be comfortable with your partner being a sperm or egg donor?	NS	*P* < 0.05*	NS	NS	NS	*P* < 0.001*	NS	NS
Would you ever consider having a biological baby using a surrogate mother?	NS	NS	NS	NS	NS	NS	*P* < 0.001*	NS
What do you suggest should be done with some of the remaining frozen embryos?	NS	*P* < 0.001*	NS	*P* < 0.05*	NS	*P* < 0.001*	NS	*P* < 0.05*
Who should have the legal power to decide on what to do with remaining frozen embryos?	NS	NS	NS	*P* < 0.001*	NS	*P* < 0.001*	NS	*P* < 0.05*
How do you think the legal status of frozen embryos should be determined?	*P* < 0.05*	*P* < 0g*P* < 0.05*	NS	*P* < 0.05*	NS	NS	NS	NS
Overall, how would you rate your current knowledge of human ART and infertility treatments?	NS	NS	NS	NS	NS	NS	*P* < 0.001*	NS

## Discussion

The findings of this study provide a comprehensive overview of the demographic characteristics, attitudes, and knowledge regarding ART among a diverse group of women in Southeast Alabama. The results highlight several key trends and offer valuable insights into the factors influencing perspectives on ART. From the time of the first successful *test tube* baby in 1978 [12] to date, medical processes addressing fertility challenges like IVF and ART continue to evoke strong public sentiment revolving around religious, moral, and ethical debates. In this study, our evaluation focused on the women’s overall awareness, prevailing attitudes, and how demographics and previous life experience influenced their views on the use of ART. The results gathered provided insight into the complexity surrounding ART and the potential barriers that influence its acceptance and utilization by women. 

Knowledge of ART 

Despite most participants (*n* = 128, 89%) reporting some level of familiarity with ART, their responses to more detailed questions highlighted a discrepancy between perceived and actual knowledge. 

These results align with prior research demonstrating that self-assessed knowledge often exceeds actual understanding, a cognitive bias well-documented in the literature. For example, while nearly half of the participants accurately identified the typical number of IVF cycles required for a successful conception (two to three cycles), only about a quarter were able to correctly estimate success rates for women over 40, with most of the women overestimating the success rates. This discrepancy suggests that, while women in this population may be somewhat familiar with the general concept of ART or IVF, they have only a superficial level of understanding and vastly overestimate its success. These findings align with previous studies, which also highlight similar misconceptions regarding IVF success rates. For example, a study by Fauser et al. [13] in Europe involving more than 6,000 people also showed over-optimism about the success of IVF. Such misperceptions may lead to women delaying childbirth, thinking that they can always rely on ART to overcome age-related fertility challenges. Current evidence suggests a general lack of awareness regarding the limitations of ART [14]. Misperceptions may be reinforced by media narratives that emphasize cases of women, particularly celebrities, giving birth at advanced ages [15]. These reports often fail to disclose that many such individuals had eggs harvested and cryopreserved at a younger age for later use. This gap in accurate reporting underscores the need for targeted educational interventions to ensure that women have access to reliable information when making reproductive decisions.

Our assessment of women’s understanding of risk factors associated with ART complications revealed that most participants demonstrated general awareness of potential maternal and fetal risks. A substantial proportion correctly identified maternal risks such as miscarriage, ectopic pregnancy, and multiple gestation. Similarly, two-thirds of participants accurately recognized birth defects and low birth weight as possible complications associated with ART.

While participants demonstrated notable awareness of maternal and fetal risks associated with ART, gaps in knowledge were evident. For instance, approximately two-thirds of respondents believed that children conceived through ART were at increased risk for conditions such as birth defects, despite this not being scientifically substantiated. Large-scale studies, including an observational study in Australia, initially reported a higher incidence of birth defects among ART-conceived children compared to those conceived spontaneously; however, after adjusting for parental factors and other confounding variables, the association was found to be statistically nonsignificant [16]. Moreover, evidence suggests that factors such as maternal and paternal age, genetics, and underlying causes of infertility contribute more substantially to the risk of birth defects than ART itself, despite public perceptions to the contrary [17].

Nearly a third of the respondents incorrectly cited Down syndrome as a risk for infants conceived through IVF. The belief may stem from the misperceptions that may arise from the well-documented association between advanced maternal age and increased risks of chromosomal abnormalities that include Down syndrome. Given that most people using ART are often at an advanced age, any increase in the occurrence of chromosomal abnormalities is then mistakenly attributed to ART when, in reality, this is an age-associated effect.

Similarly, of the 144 participants, 30 (21%) incorrectly identified autism as a risk for infants conceived through IVF. Autism has received considerable attention in recent years, with prevalence in the United States increasing by approximately 384% between 2000 and 2022 [18]. This apparent rise primarily reflects broader diagnostic criteria, improved screening and access to services, and increased public awareness, rather than a true increase in incidence. Nevertheless, social media posts and online forums frequently misattribute the high prevalence of autism to various medical interventions, including vaccines and ART. Consequently, it is understandable that many women may perceive ART as being linked to autism. However, current evidence demonstrates no association between ART and autism [19]. 

Attitudes and opinions 

The findings of this study indicate broad support for the use of ART, particularly regarding gamete donation, with 120 (83%) participants approving egg donation and 122 (85%) approving sperm donation. Despite this general endorsement, participants were less comfortable with personal involvement in donation or the use of surrogacy, reflecting a nuanced perspective in which ART is supported in principle but met with hesitation when considering direct participation. Although our study did not explore the underlying reasons for these reservations, previous research suggests that objections to donation are often driven by concerns about children being raised outside the immediate family, potential future contact with donor-conceived offspring, and fear of accidental incest [20,21].

Consistent with prior research [22,23], most women in this study identified financial costs as a significant barrier to accessing ART. Given the substantial expense associated with ART, several studies have advocated for the development of federal regulatory policies and public financing mechanisms to enhance equitable access to fertility care [23]. In this study, we sought to assess women’s perspectives on federal funding for ART-related procedures. Findings indicated strong support for federal funding for IVF in cases where individuals face medically indicated infertility, such as cancer survivors or those unable to conceive their first child naturally. In contrast, support was only moderate for funding ART for individuals seeking additional children. Notably, there was minimal support for federal funding for women who delay childbearing by choice and later require ART, suggesting a general view that public resources should not subsidize lifestyle-driven reproductive decisions. This aligns with broader evidence indicating that while empathy exists for biologically determined infertility, support diminishes when barriers to conception are perceived as voluntary or lifestyle related [13]. Furthermore, a substantial proportion of participants did not regard infertility as a health condition equivalent to other medical issues warranting federal funding, highlighting ongoing challenges in framing ART within the broader context of public health priorities.

Following the recent Alabama Supreme Court ruling that granted frozen embryos the same legal rights as children [7], an action that initially led several IVF providers to suspend services, we sought to explore women’s perspectives on this issue. Given the generally conservative context of Southern Alabama, we hypothesized that most women would support the Supreme Court’s characterization of frozen embryos as legal children. Contrary to this expectation, our data revealed low acceptance of defining frozen embryos as “children” in a legal sense, and limited support for legislative protections at all stages of embryonic development. The prevailing sentiment among participants favored minimal governmental involvement in reproductive health decisions. When asked who should determine the legal or general status of frozen embryos, most respondents preferred scientists, followed by public opinion, while very few supported decision-making by religious leaders, judges, or legislative bodies. These findings align with prior research indicating that the public tends to place greater trust in scientific expertise over political or legislative authorities when addressing health-related matters [24].

Influence of demographics and life experiences 

We examined how demographic factors and prior experiences shaped women’s perspectives on the use of ART. Our findings indicate that women with higher educational attainment expressed greater support for IVF and ART. This is consistent with a Pew Research Center survey, which revealed that higher education enhances comfort with complex medical topics, such as ART, and facilitates access to reliable information [25]. Additionally, higher educational attainment often correlates with greater socioeconomic resources, including improved health insurance, income, and healthcare access, which can mitigate barriers that commonly limit utilization of ART [26].

The observation that Christian participants reported significantly lower cumulative attitude scores compared to individuals with no religious affiliation or those of other faiths is consistent with prior scholarship demonstrating the influence of religious beliefs on reproductive decision-making and attitudes [27]. Certain Christian groups raise moral and ethical objections to ART, emphasizing the sanctity of natural conception [28]. While existing research suggests that Christians often express general support for IVF and other ART procedures, they remain less likely to utilize these technologies relative to individuals unaffiliated with religion [28]. This discrepancy between articulated support and actual utilization highlights the presence of implicit or unacknowledged biases, which may help explain the overall lower attitude scores observed among Christian participants. Interestingly, despite these patterns, most Christian respondents in this study indicated in self-reflective surveys that religion did not influence their views of ART, suggesting a possible gap between self-perceived and actual religious influence.

Our findings also revealed racial differences, with non-White respondents demonstrating more positive attitudes toward ART compared to their White counterparts. These variations may be shaped by cultural and societal norms, particularly within communities where procreation is strongly emphasized as a social expectation. For instance, research suggests that in some Hispanic and African American communities, the value placed on having biological children potentially heightens the perceived utility of ART as a means of fulfilling familial and societal obligations in the context of infertility [29]. Moreover, studies indicate that in settings characterized by traditional pronatalist values, the cultural emphasis on larger families may intersect with greater acceptance of modern medical interventions, including ART, as legitimate pathways to achieving parenthood [30].

Despite our data showing a high acceptance of use of ART among Hispanic and African American women, a previous study by Dongarwar et al. [29] showed that non-Hispanic Black and Hispanic women are less likely to receive any form of infertility treatment compared to their Caucasian counterparts. It is possible that factors such as restricted access to specialized care and the prohibitive cost of ART could be significant barriers to the use of ART in some low socioeconomic status communities. Our data would, thus, reinforce a call by Njagi et al. [23] for federal governments to devise appropriate ART regulatory policies and implement effective mechanisms for public financing of fertility care that ensure there is improved equity in access.

Historically, women in rural areas with shortages of reproductive health specialists and limited access to credible information were particularly vulnerable to ART-related misconceptions [31]. However, our study did not reveal significant urban-rural differences in knowledge, possibly reflecting the equalizing effect of widespread internet and social media access. While digital platforms can reduce informational disparities, they also pose risks, given that misinformation often spreads more rapidly than factual content, complicating public understanding of ART [32].

Experiential factors played a significant role in shaping attitudes toward ART. Participants who personally knew someone who had undergone ART or who had experienced unsuccessful pregnancies themselves expressed more favorable views of these technologies. This pattern highlights the influence of lived experiences and social networks in normalizing ART and fostering greater acceptance. Recent research similarly demonstrates that personal and vicarious experiences with infertility and fertility treatments can reduce stigma, enhance perceived legitimacy of ART, and increase willingness to pursue such interventions [33].

Study limitations 

While this study provides valuable insights into women's knowledge and attitudes toward ART in Southeast Alabama, its findings are limited by the geographic scope and relatively small sample size (*n* = 144), which may not reflect broader national or global perspectives. Our recruitment of participants mainly from an obstetric and gynecology clinic might also have caused selection bias. Furthermore, the reliance on self-reported data introduces potential biases such as social desirability. These results should, thus, be interpreted with caution and in the appropriate context.

## Conclusions

This study underscores the significance of providing women with accurate, evidence-based information on ART and how that can counter myths and enable women to make informed decisions. We conclude that adoption of proper educational strategies can enable healthcare providers to be trusted sources of information on ART while more efforts can be put to leverage digital platforms to counter misinformation, particularly in conservative or underserved settings. Future research should examine more demographic, cultural, and experiential factors shaping ART attitudes. Longitudinal studies could track how perspectives shift with evolving social and technological contexts, while qualitative approaches may illuminate the underlying values influencing reproductive decision-making.

## Appendices

Appendix

**Table 10:** Study instrument.

Question	Responses
Part 1: Demographic Data	
Which of the following ethnicity do you identify as?	Caucasian / White South Asian East Asian or Native Hawaiian or Pacific Islander African American or Black Middle Eastern North African Hispanic or Latino Other, please specify
What is your age?	16-24 25-34 35-44 45+
Have you given birth before	Yes, I have given birth before No, this is my first pregnancy No, I have been pregnant before but have not given birth No, I have never been pregnant
What is the highest level of education you have completed?	Did not complete High School High School/ GED Associate degree/Trade school Bachelor’s degree Graduate degree
Which religion do you currently practice?		Christianity Judaism Islam Buddhism Hinduism Sikhism Jainism Atheism Agnosticism Other (please specify): I do not belong to any faith tradition
*Which of the following best describes the area where you currently reside? *	Urban: A city or densely populated area. Suburban: A residential area near a city, it has a mix of housing and commercial areas Rural: A countryside area with low population density.
*Where does your estimated combined family income per annum fall?*	Under $25,000 $25,001 to $50,000 $50,001 to $75,000 $75,001 to $100,000 $100,000 to $150,000 Greater than $150,000
*Have you ever used Assisted Reproductive Technology (ART) procedures yourself?*	Yes No
Part 2: Attitudes and Perspectives of women on ART	
*What is your opinion on the use of Assisted Reproductive Technology in humans? *	Completely opposed Rather oppose Neither oppose nor support it Rather support Completely support
*Do you think that the availability of IVF and other ART encourages people to delay childbearing? *	Yes No
*Would you/ Have you ever considered using IVF treatment? Check all that apply*	Yes, I/my partner have/has had IVF treatment Yes, I/ my partner have/has considered IVF (but did not do it) Yes, I would definitely consider IVF treatment in the future, if I/my partner had fertility issues Yes, I (or wish that my partner) would consider IVF treatment in the future to start a family in later life (but we are not facing fertility issues now) e.g. social freezing No, I have never had, nor would I ever consider IVF
*Do you support the practice of egg donation?*	Yes No
*Do you support the practice of sperm donation? *	Yes No
*Under what circumstances do you think it would be appropriate for funding of In vitro Fertilization (Test-tube babies) to be provided by the Federal government (select all options that you think are appropriate) *	When having a first child and unable to conceive naturally When having another child (in addition to any they currently have) and unable to conceive naturally When a decision has been made to have a child later in life due to medical studies and other national commitments such as military deployments When fertility has been risked /sacrificed (e.g. due to cancer treatment) Federal government should never fund IVF procedure because there are other ways to have a child (for example adoption) Federal government should never fund IVF because there are better medical issues to fund from taxpayers money Funding for IVF should always be done by Individuals
*How much would you be willing to pay to have a child through Invitro Fertilization (Test-tube baby) if required? *	$0, would only do it if it is fully funded by Federal government $1 - $2500 $2600-$5000 $5001- $10 000 $10 001 - $20 000 $20 001- $25 000 $25 001 -$50 000 > $50 001 I will pay any amount to have my own child
*Would you be comfortable with your partner being a sperm or egg donor? *	Yes No
*Which of the following factors will likely influence your decision to use or not ever use Invitro Fertilization? Click all that apply.*	Cost Religion Number of desired children Health status Fertility issues
Would you ever consider having your biological baby using a surrogate mother	Yes No
Part 3: Opinions of women on Alabama’s frozen embryo ruling	
In the process of using Invitro fertilization for conception often more embryos than can be conceived are collected. These embryos are then stored in liquid nitrogen as frozen embryos which can then be transferred to the women’s uterus when conception is desired. Recently, the Alabama supreme court gave a ruling that these frozen embryos should be legally recognized as “children”.	
*To what extent do you agree with this legal ruling that classifies frozen embryos as “children”? *	Strongly agree Agree Neutral Disagree Strongly disagree
*Which of the following statements best describes the reasoning behind your acceptance or disagreement with the Alabama Supreme Court ruling (Pick all that aligns with your views) *	It aligns with my personal values and principles. My past experiences have shaped this viewpoint. I have professional expertise in this area. It conflicts with my religious beliefs I believe embryos should be recognized as children only when they have a recognizable heartbeat I believe life begins at conception, so embryos should be recognized as children once they are fertilized. I recognize frozen embryos have the potential for human life, influencing my perspective. I support the ruling as it provides legal protection for embryos and potential life. I oppose the ruling as it infringes on reproductive rights and could restrict access to ART. I am unsure about the ruling and would like to learn more before forming an opinion.
*What do you suggest should be done with some of the frozen embryos that remain after couples have had their IVF cycle completed? *	Donated for use by other couples Donated for laboratory experiments Store them for at least 10 years Just destroy them
*Who do you think should have the legal power to decide on what to do with any remaining frozen embryos? *	Couples who had the embryos frozen Laboratory where embryos are stored Physician providing IVF service State/Federal government
*Given the controversy generated by the recent Alabama Supreme Court ruling, how do you think the legal status of frozen embryos should be determined?*	Scientific consensus Public opinion Religious leadership State judges State senate and house
*To what extent would you support a law that provides legal protection for embryos, whether frozen or in the uterus, at all stages of development?*	Strongly support Somewhat support Neutral Do not support
Part 4: Knowledge of women on ART	
*Overall, how would you rate your current knowledge of human assisted Reproductive Technologies (ART) and infertility treatments *	No knowledge Some knowledge Fairly knowledgeable Very knowledgeable
*With some couples Invitro fertilization often requires several attempts (cycles) before a successful conception occurs. On average, how many cycles of in vitro fertilization (IVF) do you think a woman has to go through before a successful conception occurs?*	1 cycle 2-3 cycles 4-5 cycles 5+ cycles
*What percentage of In vitro Fertilization do you think successfully leads to a conception in women aged over 40 years? *	0-20% 21-40% 41-60% 61-80% 81-100%
*Based on your knowledge of assisted reproductive technologies, such as IVF, which of the following would you consider to be potential risks or complications for the mother? Please check all that apply: *	Miscarriage Multiple pregnancy Ectopic pregnancy Stress. IVF can be draining for the body, mind and finances Premature delivery Ovarian hyperstimulation syndrome Increased likelihood of obesity Increased likelihood of Post-partum Depression, also known as “Baby Blues”
*Based on your knowledge of assisted reproductive technologies, which of the following would you consider to be potential risks or complications for the babies conceived through IVF? Please check all that apply:*	Baby will have an increased likelihood of being born autistic Baby will have an increased likelihood of being born with Down Syndrome Baby will have an increased likelihood of developing allergies Baby will have an increased likelihood of being born with ADHD Birth defects Childhood Obesity Low birth weight
